# Altered splicing associated with the pathology of inflammatory bowel disease

**DOI:** 10.1186/s40246-021-00347-y

**Published:** 2021-07-23

**Authors:** Kiera Berger, Hari Somineni, Jarod Prince, Subra Kugathasan, Greg Gibson

**Affiliations:** 1 School of Biological Sciences and Center for Integrative Genomics, Georgia Institute of Technology, Atlanta, GA 30332 USA; 2 Department of Pediatrics, Emory University, Atlanta, GA 30322 USA; 3 Current address: insitro, San Francisco, CA 94080 USA

**Keywords:** Inflammatory bowel disease, Splicing, RNA-seq, Percent spliced in, Gene expression

## Abstract

**Background:**

Aberrant splicing of individual genes is a well-known mechanism promoting pathology for a wide range of conditions, but disease is less commonly attributed to global disruption of exon usage. To explore the possible association of aberrant splicing with inflammatory bowel disease, we developed a pipeline for quantifying transcript abundance and exon inclusion transcriptome-wide and applied it to a dataset of ileal and rectal biopsies, both obtained in duplicate from 34 pediatric or young adult cases of ulcerative colitis and Crohn’s disease.

**Results:**

Expression and splicing covary to some extent, and eight individuals exhibited aberrant profiles that can be explained by altered ratios of epithelial to stromal and immune cells. Ancestry-related biases in alternative splicing accounting for 5% of the variance were also observed, in part also related to cell-type proportions. In addition, two individuals were identified who had 284 exons with significantly divergent percent spliced in exons, including in the established IBD risk gene *CEACAM1*, which caused their ileal samples to resemble the rectum.

**Conclusions:**

These results imply that quantitative differences in splice usage contribute to the pathology of inflammatory bowel disease in a previously unrecognized manner.

**Supplementary Information:**

The online version contains supplementary material available at 10.1186/s40246-021-00347-y.

## Background

Defective RNA splicing contributes to the etiology of a wide variety of diseases [1]. Single gene defects that weaken or abolish splice sites or activate cryptic ones have been associated with over 200 human diseases, including progeria, cystic fibrosis, muscular dystrophies, and some cancers [2–5]. Computational analyses have further identified variants in over 80,000 splicing regulatory motifs [6], and scores such as TraP (TRanscript inferred Pathogenicity Score) provide pre-computed predictions of likely splice defects for polymorphisms affecting all human genes [7]. Just as importantly, global mis-regulation of the splicing of hundreds of genes due to aberrant activity of components of the spliceosome, is known to contribute to pathology for a variety of conditions, notably myelodysplastic syndrome, myotonic dystrophy, several neurological disorders, and cancer metastasis [8–12].

The inflammatory bowel diseases (IBD), ulcerative colitis (UC), and Crohn’s disease (CD) afflict approaching 1% of adults in developed countries and have been rising in prevalence globally for several decades [13]. They are well-known to involve aberrant gene expression in the gut [14, 15] as well as peripheral immune system [16, 17], and signatures of severe disease at diagnosis have been associated with progression to complicated disease or remission [18–20] and are being developed as biomarkers of therapeutic response [21]. There is also some indication that gene expression is to some extent ancestry-dependent, resulting in mis-regulation of pathways related to cytokine signaling, extracellular matrix function, and mitochondrial activity that is biased toward more adverse outcomes in African Americans [22]. To date, to our knowledge, there have not been any reports of splicing defects in IBD, so we asked whether transcriptome profiles assessed by the RNA-seq of bulk ileal and rectal tissues might provide evidence for unusual splice isoforms associated with IBD in a dataset of paired ileal and rectal biopsies from a cohort of 34 young individuals with CD or UC.

## Results

### Effects of disease, location, and ancestry on splicing and gene expression

In order to quantify the influences of disease, location, and ancestry on splicing and gene expression, we first computed the principal components (PC) for both the transcript abundance and PSI (percent spliced in) counts from the RNA-seq dataset and then generated a weighted sum of the influences on these measures. This principal component variance analysis revealed that three quarters (75%) of the expression variability and one third (33%) of the splicing variability was captured by the first ten principal components of the respective measures, indicating that gene level expression is far more variable than exon usage between rectal and ileal tissue. For gene expression, 40.3% of the variance was between locations (ileum and rectum), 2.3% between ancestry groups (European and African), 0.2% between disease subtypes (UC and CD), and 0.8% captured by the interaction between location and disease. Corresponding percentages for the splicing variance were 20.7% between locations, 5.8% ancestry groups, 0.7% disease, and 0.8% the interaction effect. These proportions and the contributions to each PC are provided in Table 1, which also shows that the variance contributions to PSI are relatively unaffected by the threshold of inclusion, being similar for datasets with 108,091 or 7001 exon bins. In both cases, then, consistent with previous studies, by far the largest effect is between ileum and rectum [23], a meaningful ancestry component is observed [22], and twice as much variance is due to the differences in the effect of disease on the two tissues than to disease across both tissues.

**Table 1:** Principal component variance analysis (PCVA) decomposition of sources of variance

	Gene expression	PSI (108,091 exon bins)	PSI (7001 exon bins)
Principal component			
PC1	28.9%	7.6%	9.3%
PC2	25.1%	5.5%	6.9%
PC3	5.2%	4.1%	4.1%
PC4	4.4%	3.0%	3.2%
PC5	3.5%	2.6%	2.9%
PC6	2.3%	2.4%	2.7%
PC7	2.0%	2.3%	2.3%
PC8	1.6%	1.8%	2.0%
PC9	1.6%	1.7%	1.9%
PC10	1.3%	1.7%	1.6%
SUM of PC1–PC10	75.9%	33.0%	27.9%
Variance component			
Ancestry	2.3%	5.8%	5.3%
Location	0.2%	0.7%	0.5%
Disease	40.3%	20.7%	27.9%
Location*disease	0.8%	0.8%	1.0%

The table shows the percent variance in gene expression or splicing (PSI at two inclusion thresholds) explained by the first ten PCs and their sum, as well as the weighted contribution of each variance component term (ancestry, disease, location, and the interaction of disease and location) to these 10 PCs. Gene expression is for 18,929 genes, and number of PSI bins is before and after the final two stages of filtering (*n* = 119 samples for all)

At the 5% False Discovery Rate, there were 9569 differentially expressed genes by location, 1847 by ancestry, and just 556 by disease, although 1570 showed an interaction effect, implying that most disease effects, as expected, are specific to the ileum (in CD) or rectum (in UC). Correspondingly, there were 1885 significant PSI by location (listed in Table S1), 90 by ancestry, and none by disease or showing an interaction effect, implying that disease has a much smaller impact on splicing in each tissue than it does on overall expression.

**Fig. 1:**
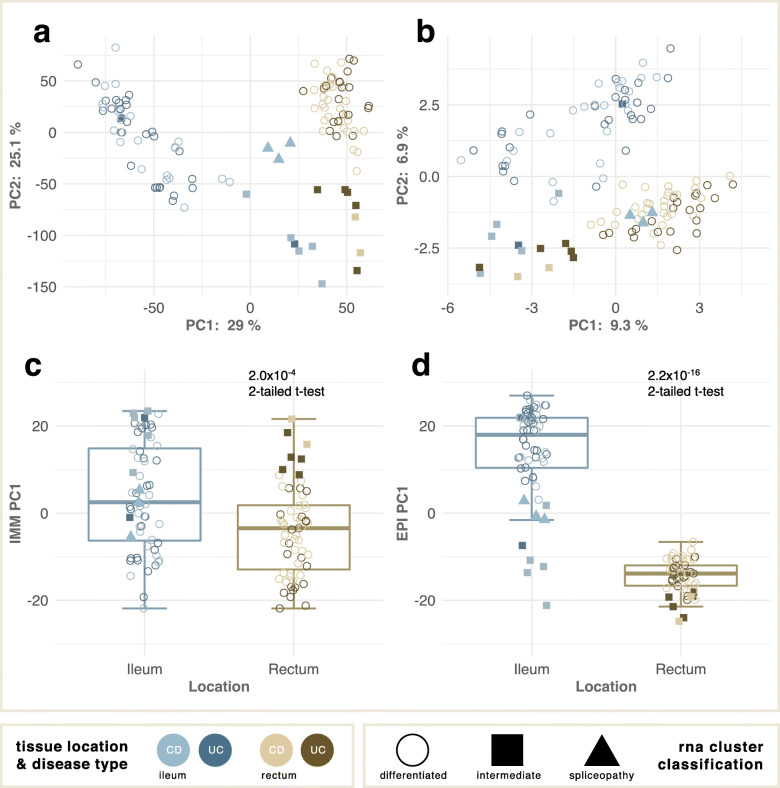
Principal components of transcript variation. **a** PC2 vs PC1 of transcript abundance showing separation of ileal (blue, *n* = 60) and rectal (brown, *n* = 59) samples along PC1, and of intermediate samples (solid squares) along PC2. Six of 8 intermediate individuals are represented by two samples each; different individuals are intermediate in the two tissues. **b** PC2 vs PC1 of exon usage (PSI) showing similar separation by tissue, but with three ileal samples (blue triangles) clustering with the rectal set. Percentages refer to variance explained, shading to disease status. **c**, **d** Differential abundance of immune (**c**) and epithelial (**d**) cell contributions summarized by PC1 of compartment-specific gene expression differentiate ileum and rectum

The first principal component (PC1) of gene expression and the first two principal components (PC1 and PC2) of splicing provide particularly strong separation by location as seen in Fig. 1a,b, respectively, with the exception of samples from 8 individuals highlighted by the solid squares which are also extreme for PC2. We provide evidence in Fig. 1c,d and Fig S1 that these major components of variation reflect the proportions of the three major tissue compartments [24, 25], specifically with elevated epithelial contribution to the ileum relative to the rectum and immune and fibroblast contributions to the intermediate samples. Analysis of the genes altered in the intermediate-type ileal and rectal cases indicate that the differentiation of these samples is likely driven by an amplified immune response. Of note, the IBD-associated genes *TLR2*, *TLR4*, and *NOD2* exhibit elevated expression in intermediate ileal and intermediate rectal samples.

### Aberrant profiles define “spliceopathies”

**Fig. 2:**
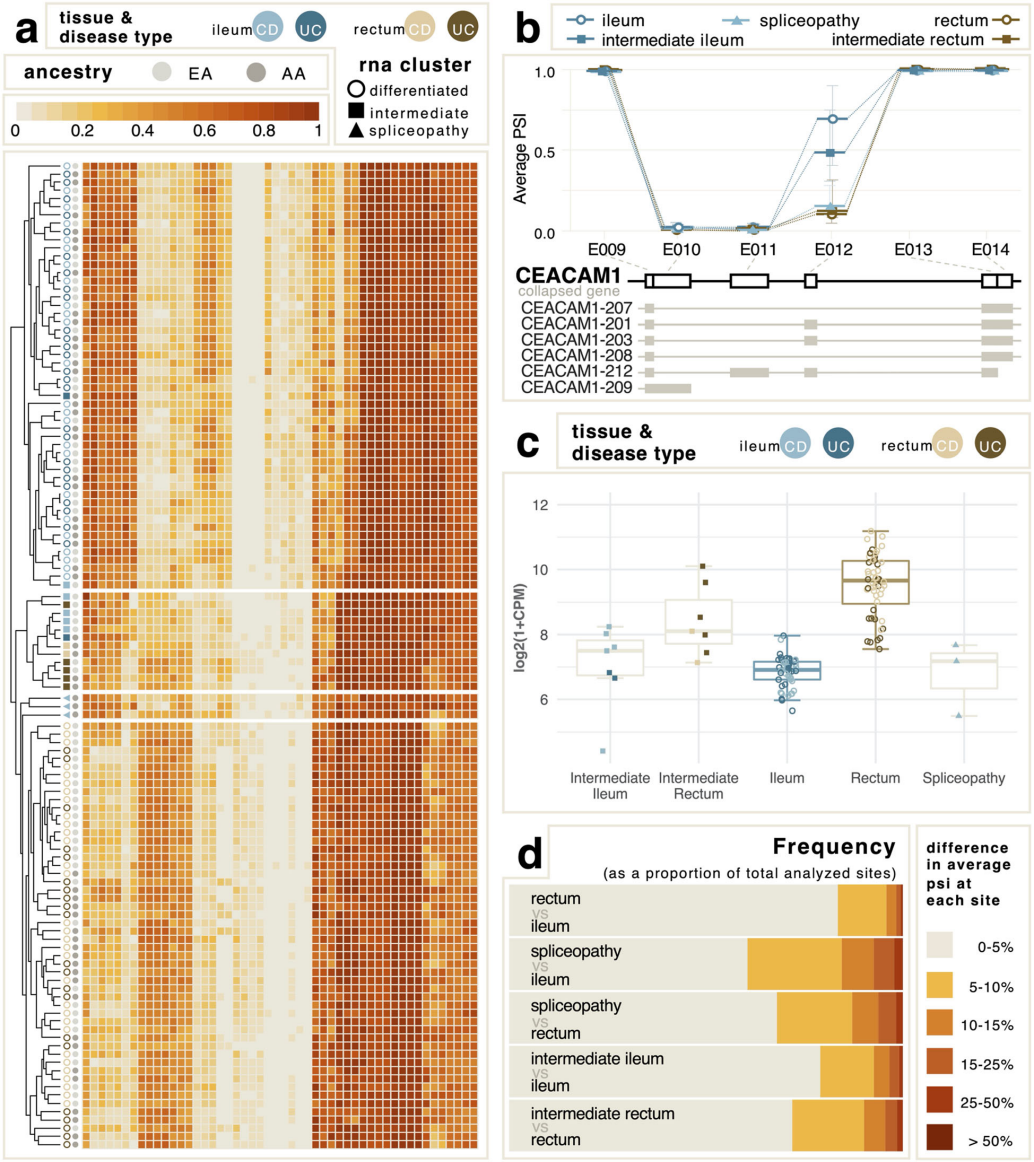
Characteristics of the spliceopathy samples. **a** Heat map of the top 50 most differentially abundant exons showing broad clustering by tissue (rectum to the left) but not disease status. The intermediate samples cluster as a group, adjacent to the ileal spliceopathies which are closer to the rectal set and include aspects of both tissues. **b** Average PSI of exon bins 9 through 14 of *CEACAM1*, showing average levels of E012 (corresponding to *CEACAM1* exon 7) differ by tissue and state. **c** Gene expression of *CEACAM1* by cluster. Intermediate ileum and spliceopathy samples are not significantly different from differentiated ileum, whereas intermediate rectum and differentiated rectum are both significantly elevated relative to ileum. **d** The proportion of sites with indicated difference in average PSI for comparisons of ileum (*n* = 50) to rectum (*n* = 52), spliceopathy ileum (*n* = 3) to both ileum and to rectum, and intermediate ileum (*n* = 7) or rectum (*n* = 7) to corresponding differentiated tissue. The most differential splicing is observed in each bin above 5% for the spliceopathies

Three ileal samples highlighted by the solid blue triangles in Fig. 1a,b (2 from one donor, 1 from another who did not have a paired ileal biopsy) have rectal-like splicing yet gene expression intermediate between the ileum and rectum (see also Fig S1). These two Crohn’s disease cases thus have particularly altered splicing, suggesting that their disease is due to a “spliceopathy”. Analysis of variance detected 284 differentially used splice sites in the three samples compared with the ileum (Table S2), and the heat map in Fig. 2a highlights how these ileal samples globally more resemble the rectum in terms of exon usage. These differentially used splice sites come from 246 genes, of which only 104 were found to be differentially expressed at the gene level, further supporting the suggestion of a “spliceopathy”. A representative example, *CEACAM1,* itself an established IBD risk gene [26] whose product regulates mucosal inflammation via T-cells [27], is shown in Fig. 2b where exon bin 12 has low, rectal-like PSI in the ileum, whereas the other intermediate samples are more ileal-like. The overall expression of the gene is normal (Fig. 2c).

There are two main isoform types of *CEACAM1* that differ in the length of the cytoplasmic tail. The inhibitory functions of the long cytoplasmic tail isoform (*CEACAM1-L*) are well studied, and *CEACAM1-L* is known to be the predominantly expressed isoform in human lymphocytes. Though *CEACAM1-S* functions are less well characterized, it has been linked to mucosal immune regulation and recent studies show that intestinal T cells primarily express this isoform [28]. Exon bin 12 corresponds to exon 7 of the *CEACAM1* gene, which is included only in the *CEACAM1-L* isoform and also contains regions involved in the alternative splicing of this gene [26, 29]. Analysis of this region did not identify any SNPs that may lead to the differential isoform ratio observed in these samples. While no other CEACAM family members exhibited altered splicing profiles, we did observe elevated gene expression of *CEACAM5,* known to be a marker of Crohn’s disease, in the spliceopathy samples [30]. However, expression was consistent with the level seen among rectal samples, further supporting the hypothesis of a transcriptome-wide defect causing these ileal samples to resemble rectal tissue.

Underscoring that the defective splicing is transcriptome-wide, Fig. 2d shows the fraction of exons in bins of differential usage for various contrasts, with the greatest deviations seen for the spliceopathy samples. Despite the widespread nature of the defective splicing, separation of samples by tissue type using PCA (principal component analysis) could also be performed reliably using just 96 of the 284 splice sites that were also differentially used in the rectal samples compared with the ileum, but did not distinguish the spliceopathy samples and rectal samples, making these exonic bins “rectal-like”. Gene ontology analysis of the genes encompassing these exons identified an enrichment of genes involved in fructose catabolism. Three out of five genes in the pathway (*KHK*, *TKFC*, and *GLYCTK*) had exons exhibiting rectal-like splicing in the ileal spliceopathy samples. In addition to differential isoform usage, overall transcript abundance of ketohexokinase (*KHK*) and triokinase (*TKFC*) was also reduced in the spliceopathy samples, to a level intermediate between the rectum and ileum. Breath testing has been used to demonstrate that fructose malabsorption is quite common in individuals with ileal Crohn’s disease [31], consistent with the hypothesis that an excess of short chain carbohydrates may be a trigger for pathogenesis.

Expansion of the gene ontology analysis to genes encompassing all 284 exons found to be differentially used in spliceopathy compared to ileum also identified enrichment of RNA splicing and spliceosome processes, suggesting that the rectal-like splicing observed in the spliceopathies is driven by an unknown aberration in the mRNA processing mechanisms of these patient’s ileal tissue. However, there was no evidence from splicing, expression, or genotype data for the involvement of any of the three RNA-binding proteins known to influence alternative splicing of CEACAM1 [29]. Two-way hierarchical clustering of PSI for the top 24 most spliceopathy-affected exons from 17 splicing-related genes in Fig S2 shows that the two samples from one individual are clear outliers, while the single aberrant biopsy from the second individual falls within a small cluster of rectal-like ileal samples. These two cases thus likely have different genetic etiologies. It is not possible from this dataset to discern whether a single mutation is responsible for the profiles, or whether a combination of genetic and environmental factors lead to disruption of the splicing of these gene products, which then mediates the broader set of aberrant splice events.

## Discussion

**Fig. 3:**
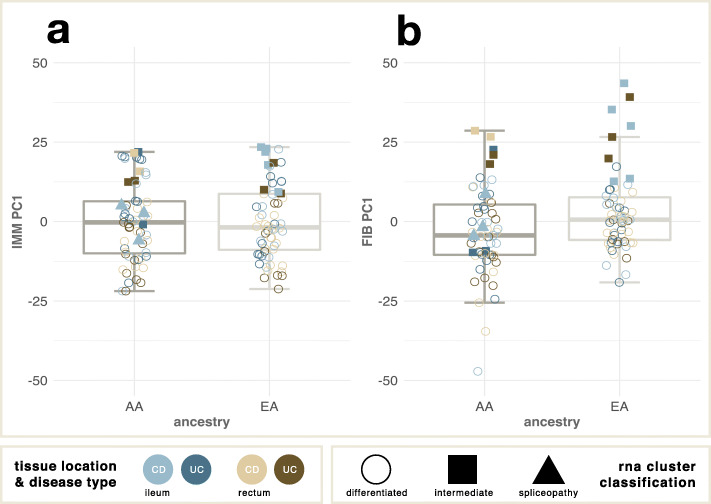
Association of ancestry with tissue proportion in biopsies. **a** PC1 of immune-specific expression is similar between the two ancestry groups. **b** PC1 of fibroblast-specific expression is significantly different between the two ancestry groups, implying a reduced proportion of fibroblasts in the African ancestry biopsies (*p* = 0.005, 2-tailed *t* test). Note that the aberrant intermediate samples have particularly elevated fibroblast expression in both groups, whereas the two “spliceopathy” cases, both African American, have relatively normal fibroblast proportions

Our results establish that altered splicing is a relevant feature of the IBD gut. Since splicing is to some extent co-regulated with transcription [1, 32], covariation of both aspects of gene expression is observed, for example in similarity of the principal components. An appreciable fraction of individuals have more rectal-like ileal expression and splicing also because of alterations in the proportions of epithelial, stromal, and immune cells. These differences are to some extent ancestry-biased, notably with elevated stromal (fibroblast) expression in Europeans relative to African Americans (Fig. 3.a,b). This observation extends our recent demonstration of ancestry-related differences in ileal gene expression involving pathways that also associate with disease severity [22]. Our ability to determine the cause and observe the downstream effects of the “spliceopathy” is limited by both the low number of individuals it was observed in and the design of our study. Future research could shed more light on the frequency, effects, and possible cause of this type of aberration by analyzing single-cell RNA-seq and variants from whole genome sequencing in addition to bulk mRNA-seq in the rectal and ileal tissues. It will be important to define the molecular mechanisms responsible for the coordinated splicing defects and to evaluate whether they suggest personalized therapeutic interventions.

A further noteworthy aspect of this study is the development of a pipeline for quantitative analysis of splicing data from RNA-seq. The popular MISO (Mixture of Isoforms) protocol [33] incorporates fragment length distributions and exon-level abundance estimates into probabilistic estimation of altered isoform usage, but is intended for single sample comparisons. Several other existing approaches to detection of aberrant exon usage are incorporated into standard RNA-seq analysis tools [34–36], while an approach based on identification of novel or cryptic splice junctions in cases compared with controls, led to identification of the molecular basis for 17 of 48 previously undiagnosed neuromuscular disease cases [37]. Here we combine attributes of each of these algorithms along with quantitative evaluation of exon usage to identify suites of concurrent aberrant splicing in outlier individuals. PSI filtering (see Methods) allows for a focus on exons that are not constitutively expressed and therefore contribute to differential isoform usage, without being limited to annotated isoforms. Similar conclusions were observed at a variety of thresholds of inclusion, but careful filtering to rule out artefacts of low expression or exon coverage allowed us to focus on a core set of a few hundred genes perturbed in two cases of spliceopathy.

This study was performed using whole mRNA, which has long been the standard for gene expression analysis and, by extension, exon level and splice site analysis. However, several aspects of whole mRNA sequencing are not ideal for analysis of these smaller features, and a case can be made for targeted RNA-seq when at all possible. Our samples, following Illumina and ENCODE recommendations [38], had a median sequencing depth of 45 million reads. While this provides robust analysis at the gene level, it is quite limiting in terms of how many splice sites can be evaluated with accuracy. After removing exonic bins in genes with low or no coverage (60% of sites), the mean number of informative reads per bin for any given sample is 169, while the average maximum number of reads at a single site is over 750,000. At over half of the remaining sites, the median PSI score across all samples is 0.99, rendering those sites uninformative for differential usage analysis. A careful review of literature and public RNA-seq databases such as GTEX could identify genes that, though highly expressed in the target tissue, are not relevant for the proposed analysis. By using this information to create a targeted RNA-seq panel, researchers can achieve a higher read depth for a more robust analysis of splicing without needing to increase overall sequencing depth or sacrifice gene level analysis.

## Conclusions

Consistent with previous studies, we found tissue location to be the largest contributor to variability in gene expression and splicing. Though gene expression differences between tissues are often accompanied by changes in splicing, as one might expect since different cell types may utilize different isoforms, neither analysis shows the whole picture on its own. The observation of the ileal samples in two CD patients exhibiting intermediate gene expression but clear rectal-like splicing indicates that differential splicing is a previously unrecognized contributor to IBD disease pathology. Because the aberrations are seen in the full splicing profile rather than a specific aberrant splicing event, we refer to these cases as a “spliceopathy”. Our results indicate that inclusion of splicing analysis when performing RNA-seq experiments for the study of human disease could play an important role in identifying additional contributions to the pathology of not only IBD, but also other complex diseases.

## Methods

We analyzed whole mRNA sequencing profiles of 124 samples obtained from 34 young donors with IBD (age range 8–20 years). Duplicate biopsies of both the ileum and rectum were analyzed, in general, 4 samples per donor, although 6 donors were represented by only 3 samples and three by a single biopsy from each location. Individuals were closely matched for ancestry (18 European, 16 African American), sex (16 male, 18 female), disease type (20 Crohn’s disease, CD; 14 ulcerative colitis, UC), and disease status at time of sampling (20 established cases, 14 cases at diagnosis). All donors were tumor free at biopsy. Following quality control, total transcript abundance was measured for 18,929 genes, and percent-spliced-in (PSI) estimates [39] were obtained for 7001 variable exon bins.

### Sequencing

RNA was extracted, and library preparation was performed using the Illumina TruSeq Stranded mRNA kit. Paired-end 100bp stranded sequencing was performed for all samples on an Illumina HiSeq at a median read depth of 22.7 million (range: 10.2–106 million) read pairs.

### Preprocessing

FastQC was run on raw fastq files to ensure mean phred scores per sequence and per base were above 27, to check consistency among samples in per sequence GC content, per base N content, sequence length distribution, and sequence duplication levels, and to check for the presence of adapters [40]. Samples were trimmed up to but not beyond the adapter using trimmomatic [41]. Samples were aligned with the STAR splice aware aligner to hg38 using the Gencode v29 primary assembly sequence and annotation [42, 43]. Default parameters were used with the following exceptions: to increase accuracy of splice site mapping and discovery, two-pass mode was invoked; novel splice junctions were required to have a minimum overhang of 8 bp, and a minimum of 5 unique reads was required for a splice site to be included in the splice junction output. To ensure each read used in downstream analysis was accurately mapped, and results were not affected by high homology regions such as pseudogenes, all multimapping reads (which map equally well to two locations in the genome) were filtered out. We further confirmed that all reads aligning to the CEACAM1 alternative splice bin did not align to the duplicate pseudogene [44], which possesses sufficiently divergent nucleotide sequence to prevent multi-mapping.

### QC

In order to remove samples exhibiting extreme 5’ or 3’ bias or mapping issues that could affect splice calculations, sample quality was assessed using the Quality of RNA-Seq Tool-set (QoRTs) which evaluates cumulative gene diversity, gene-body coverage, and number of observed splice junction loci [45]. One rectal sample from individual 6 and one from individual 26 were observed to be extreme outliers in 3’ bias and were removed, leaving both individuals with two ileal and one rectal samples. To confirm that each sample from an individual was indeed the same individual, variant calling was performed at Purcell’s 5k sites following GATK best practices and identity-by-descent was compared using output from PLINK [46–48]. A PI_HAT minimum threshold of 0.7 was used to confirm a match between two samples. The single rectal sample from individual 18 failed the identity-by-descent QC measure, leading to the removal of all samples from individual 18.

### Gene expression analysis

Overall differential gene expression was performed with DESeq2, using the STAR raw read counts per gene output and including ancestry, disease, location, and the interaction of disease and location in the design formula [49]. Prior to analysis, genes were filtered for mean coverage > 5 reads and both principal component analysis (PCA) and principal component variance analysis (PCVA) were performed on the final set of 18,929 genes, with results listed in Table 1. PCA captures the major components of covariance of gene expression, and PVCA sums the amount of variance in each PC that is associated with the influencing factor, weighted by the variance in gene expression explained by the PC. We only analyzed the first 10PC of both RNA abundance and PSI since smaller PC explained less than 1% of the variance each and tend to capture differences among individuals or noise.

Gene expression was also used to estimate abundance of specific cell types. Lists of genes expressed in immune, epithelial, and fibroblast cells were created from single-cell RNA-seq data for these cell types obtained from the colonic mucosa of ulcerative colitis patients [25], using thresholds of > 5 counts per million (CPM) in one cell type and < 1 CPM in the other two cell types. We then generated PC1 for each list and estimated the correlation with location and ancestry in order to evaluate the contribution of cell type abundance to these effects.

### Splicing analysis

Splicing patterns between individuals were compared using the percent spliced in (PSI) metric, which was calculated per exonic bin for each sample. PSI is independent of library size and yields a score between 0 and 1 representing the proportion of isoforms that include a particular exonic bin. Inclusion (IR) and exclusion (ER) read counts were obtained following the protocol outlined in Schafer et al. [39] using the splice junction output from the STAR alignment, and the recommendation of requiring > 10 ER to identify alternatively spliced exon bins was used to inform the following filtering steps. For each sample, if a site had < 10 ER the PSI score was rounded up to the nearest tenth (IR > 10) to lessen the impact of low exclusion counts or NA (IR < 10) to indicate no coverage, allowing more exonic bins to be evaluated across all samples without low ER counts dominating the analysis. To limit analysis to genes expressed in both tissues, rows where one or more samples had no coverage were excluded (511,191 exon bins, leaving 108,091). Rows where all samples had the exact same PSI (0 or 1) were removed (64,129), reducing analysis to only those sites where one or more samples had variability in level of exon exclusion. Subsequently, to focus on splice bins with potential group-wise differences, further filtering was performed to exclude splice bins where 40% of samples were close to constitutively included or excluded (> 95% and < 5% PSI, respectively). This filtering reduced the original 619,282 potential splice sites to 7001 in the final analysis, with a mean PSI score of 0.45. PCA and PCVA were performed on the PSI estimates before and after the final stage of filtering, yielding very similar results presented in Table 1. To identify differentially used exonic bins, linear mixed models were performed on the final set of PSI scores with the lme4 R package including fixed effects of disease, location, and ancestry and the interaction between disease and location, and a random effect of individual [50].

### Characterization of differential splicing in the spliceopathy samples

A heatmap of 50 exon bins (Fig. 2a) was created to visualize differences in the splicing patterns of sample groups. Because the largest contributor of variance in PSI was tissue location, the 50 most significant exonic bins by location obtained from the previously described analysis were used. Hierarchical clustering of samples for the 50 exonic bins was performed using the Euclidian distance and complete linkage method.

The difference between two PSI averages for each site was again used to observe the extent of variation between groups (Fig. 2d). Samples were split into ileum, rectum, intermediate ileum, intermediate rectum, and spliceopathy, and the difference in average PSI for each comparison was categorized at every exonic bin in the filtered 7001 exonic bins used for analysis.

For the identification of PSI sites in the spliceopathy samples that were significantly different from the differentiated ileum, the differentiated rectum, or both, exonic bin filtration criteria were relaxed slightly. To limit analysis to genes expressed in all three groups, rows where more than five ileal samples, five rectal samples, or one spliceopathy sample had no coverage were removed. The missing values in rows where 1–5 ileal or rectal samples had no coverage were replaced with the tissue location average at that site, allowing these additional 27,514 exonic bins to potentially be included in this analysis. The remainder of the filtration steps was carried out as before, this time reducing the original 619,282 potential splice sites to 9499 in the final analysis, with a mean PSI score of 0.43. To identify differentially used exonic bins, linear mixed models were performed on the final set of PSI scores including fixed effects of group (ileum, rectum, or spliceopathy) and ancestry and a random effect of individual.

## Supplementary Information

**Additional file 1: Table S1.** PSI significant by tissue location

**Additional file 2: Table S2.** PSI significant in spliceopathy compared to ileum

**Additional file 3: Figure S1.** Correlations of gene expression with specific cell type proportions. **(a,b)** Gene expression correlation with PC1 of immune-cell specific genes implying elevated immune cell proportion in the intermediate outlier samples. **(c,d)** Correlation with PC1 of epithelial-cell specific genes, emphasizing the strong contribution of these cells to the sample location differentiation. **(e,f)** Correlation with PC1 of fibroblast specific genes, showing the same elevation in proportions as immune-specific genes

**Additional file 4: Figure S2.** Aberrant splicing of splicing mediators. The heatmap shows the percent spliced in proportions for 24 exons transcribed from 17 genes annotated to regulation of splicing processes. Rows are samples, and columns PSI scores. Blue and brown shapes to the left indicate the tissue (blue, ileum; brown, rectum) next to grey circles showing ancestry (dark shade African, light European). Two spliceopathy samples from individual 5 are at the top and are outliers in the two-way hierarchical clustering; the third sample is 28A, also indicated by a triangle shape

## Data Availability

The data discussed have been deposited in NCBI's Gene Expression Omnibus and are accessible through GEO Series accession number GSE158952. Relevant code can be accessed at the following Github repository: https://github.com/kiera-gt/RectumIleumIBD-RNAseq.
