# Supplementary figures and images for: Altered splicing associated with the pathology of inflammatory bowel disease
Source: Hum Genomics. 2021 Jul 23;15:47. doi: 10.1186/s40246-021-00347-y (PMC8305504; doi:10.1186/s40246-021-00347-y)

**a**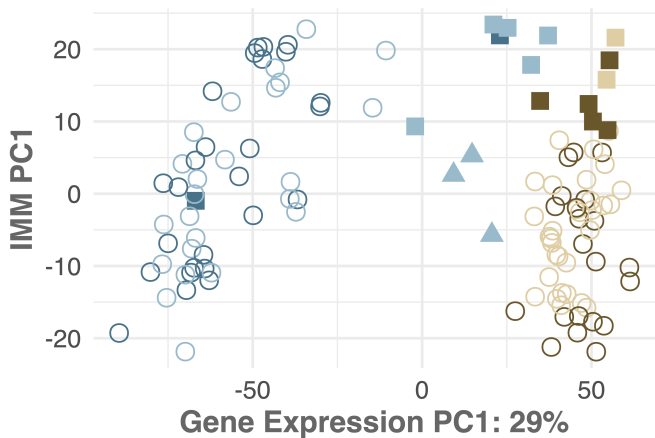**b**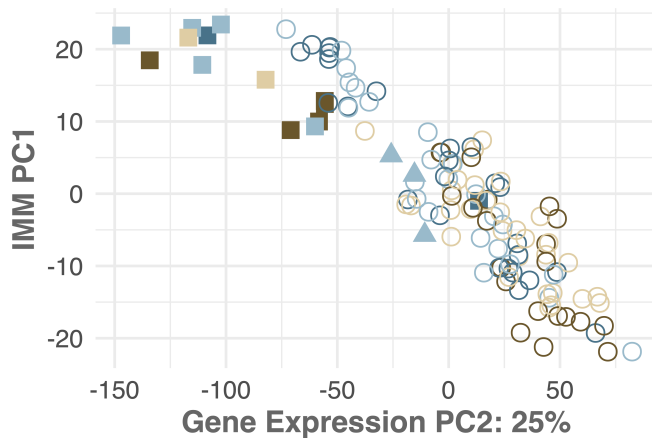**c**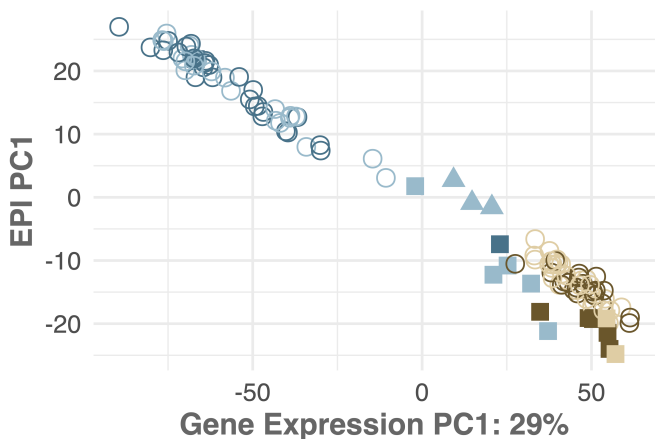**d**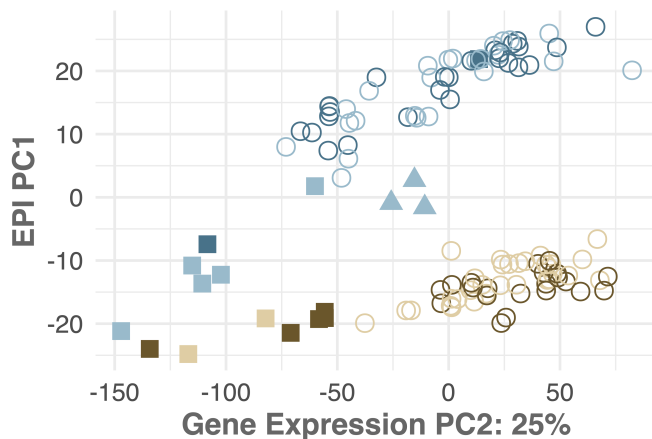**e**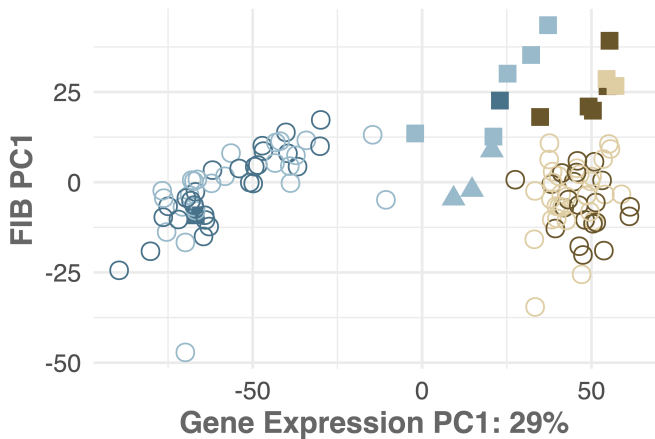**f**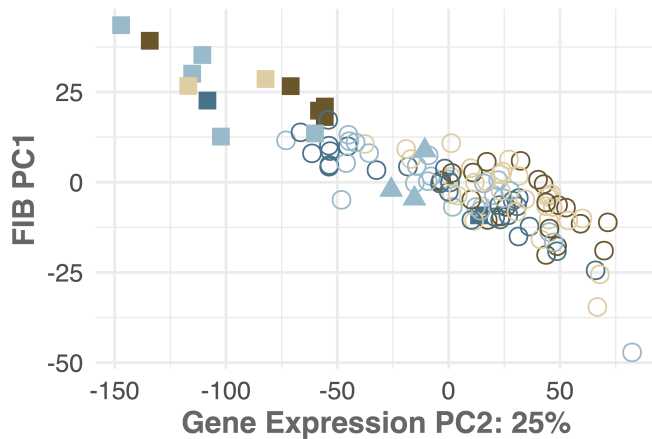

**tissue location  
& disease type**

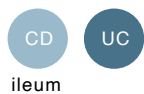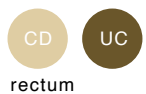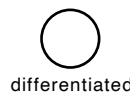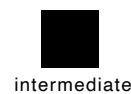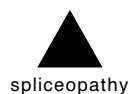

**rna cluster  
classification**

Supplement: Supplementary file 3 — Additional file 3: Figure S1. Correlations of gene expression with specific cell type proportions. (a,b) Gene expression correlation with PC1 of immune-cell specific genes implying elevated immune cell proportion in the intermediate outlier samples. (c,d) Correlation with PC1 of epithelial-cell specific genes, emphasizing the strong contribution of these cells to the sample location differentiation. (e,f) Correlation with PC1 of fibroblast specific genes, showing the same elevation in proportions as immune-specific genes [file 40246_2021_347_MOESM3_ESM.pdf]

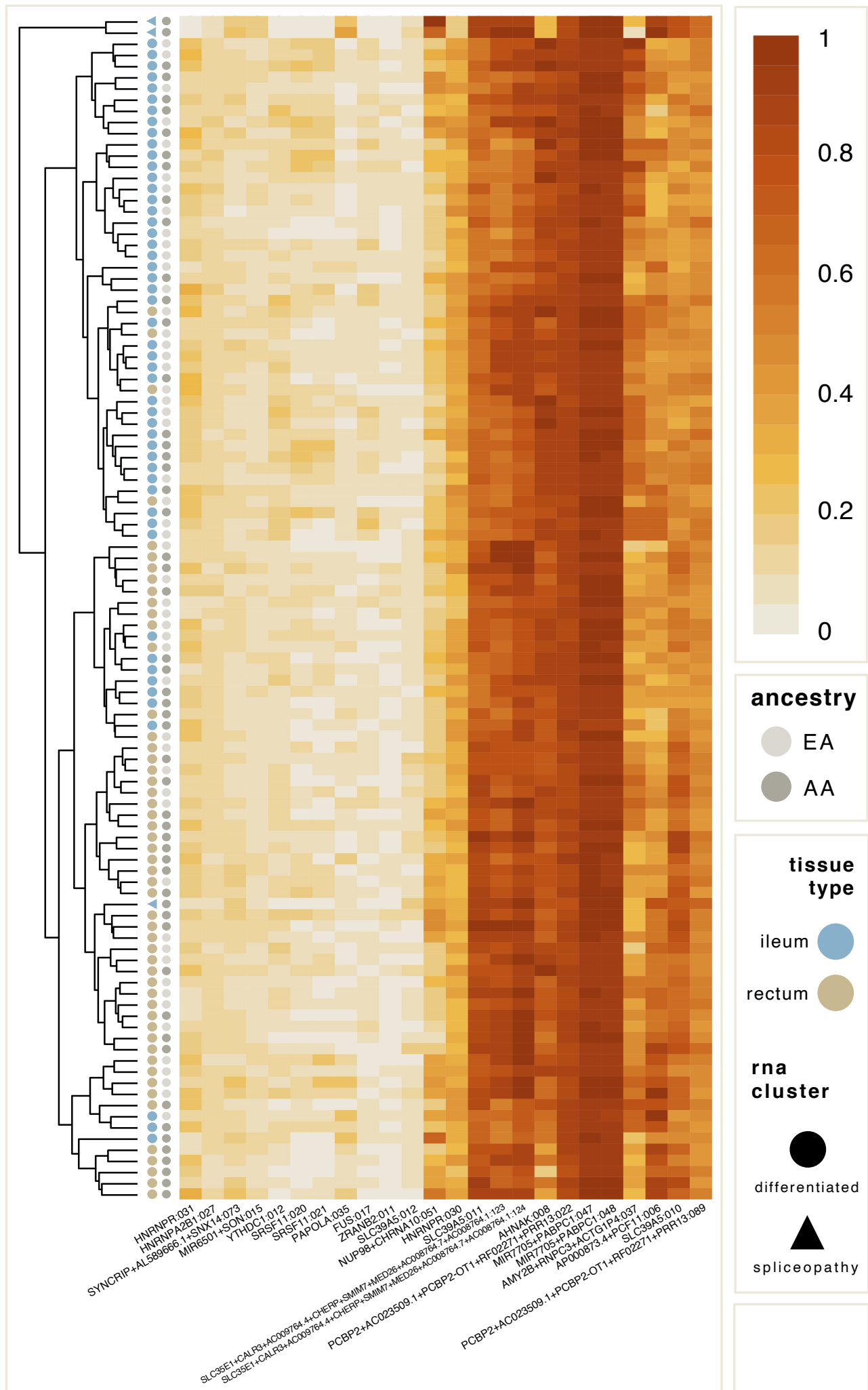

Supplement: Supplementary file 4 — Additional file 4: Figure S2. Aberrant splicing of splicing mediators. The heatmap shows the percent spliced in proportions for 24 exons transcribed from 17 genes annotated to regulation of splicing processes. Rows are samples, and columns PSI scores. Blue and brown shapes to the left indicate the tissue (blue, ileum; brown, rectum) next to grey circles showing ancestry (dark shade African, light European). Two spliceopathy samples from individual 5 are at the top and are outliers in the two-way hierarchical clustering; the third sample is 28A, also indicated by a triangle shape [file 40246_2021_347_MOESM4_ESM.pdf]
